# A hybrid multi-panel image segmentation framework for improved medical image retrieval system

**DOI:** 10.1371/journal.pone.0315823

**Published:** 2025-02-20

**Authors:** Faqir Gul, Mohsin Shah, Mushtaq Ali, Lal Hussain, Touseef Sadiq, Adeel Ahmed Abbasi, Mohammad Shahbaz Khan, Badr S. Alkahtani

**Affiliations:** 1 Department of Computer Science & IT, Hazara University, Mansehra, Pakistan; 2 Department of Telecommunication, Hazara University, Mansehra, Pakistan; 3 Department of Computer Science & IT, Neelum Campus, The University of Azad Jammu and Kashmir, Muzaffarabad, Azad Kashmir, Pakistan; 4 Department of Computer Science & IT, King Abdullah Campus, The University of Azad Jammu and Kashmir, Muzaffarabad, Azad Kashmir, Pakistan; 5 Centre for Artificial Intelligence Research (CAIR), Department of Information and Communication Technology, University of Agder, Grimstad, Norway; 6 Central South University, Changsha, Hunan, China; 7 Children’s National Hospital, Washington, DC, United States of America; 8 Department of Mathematics, King Saud University, Riyadh, Saudi Arabia; National Textile University, PAKISTAN

## Abstract

Multi-panel images play an essential role in medical diagnostics and represent approximately 50% of the medical literature. These images serve as important tools for physicians to align various medical data (e.g., X-rays, MRIs, CT scans) of a patient into a consolidated image. This consolidated multi-panel image, represented by its component sub-images, contributes to a thorough representation of the patient’s case during diagnosis. However, extracting sub-images from the multi-panel images poses significant challenges for medical image retrieval systems, especially when dealing with regular and irregular image layouts. To address these challenges, this paper presents a novel hybrid framework that significantly enhances sub-image retrieval. The framework classifies medical images, employs advanced computer vision and image processing techniques including image projection profiles and morphological operations, and performs efficient segmentation of various multi-panel image types including regular and irregular medical images. The hybrid approach ensures accurate indexing and facilitates fast retrieval of sub-images by medical image retrieval systems. To validate the proposed framework, experiments were conducted on a set of medical images from publicly available datasets, including ImageCLEFmed 2013 to ImageCLEFmed 2016. The results show better performance compared to other methods, attaining an accuracy of 90.50% in image type identification and 91% and 92% in regular and irregular multi-panel image segmentation tasks, respectively. By achieving accurate and efficient segmentation across diverse multi-panel image types, our framework demonstrates significant potential to improve the performance of medical image retrieval systems.

## 1. Introduction

**Fig 1:**
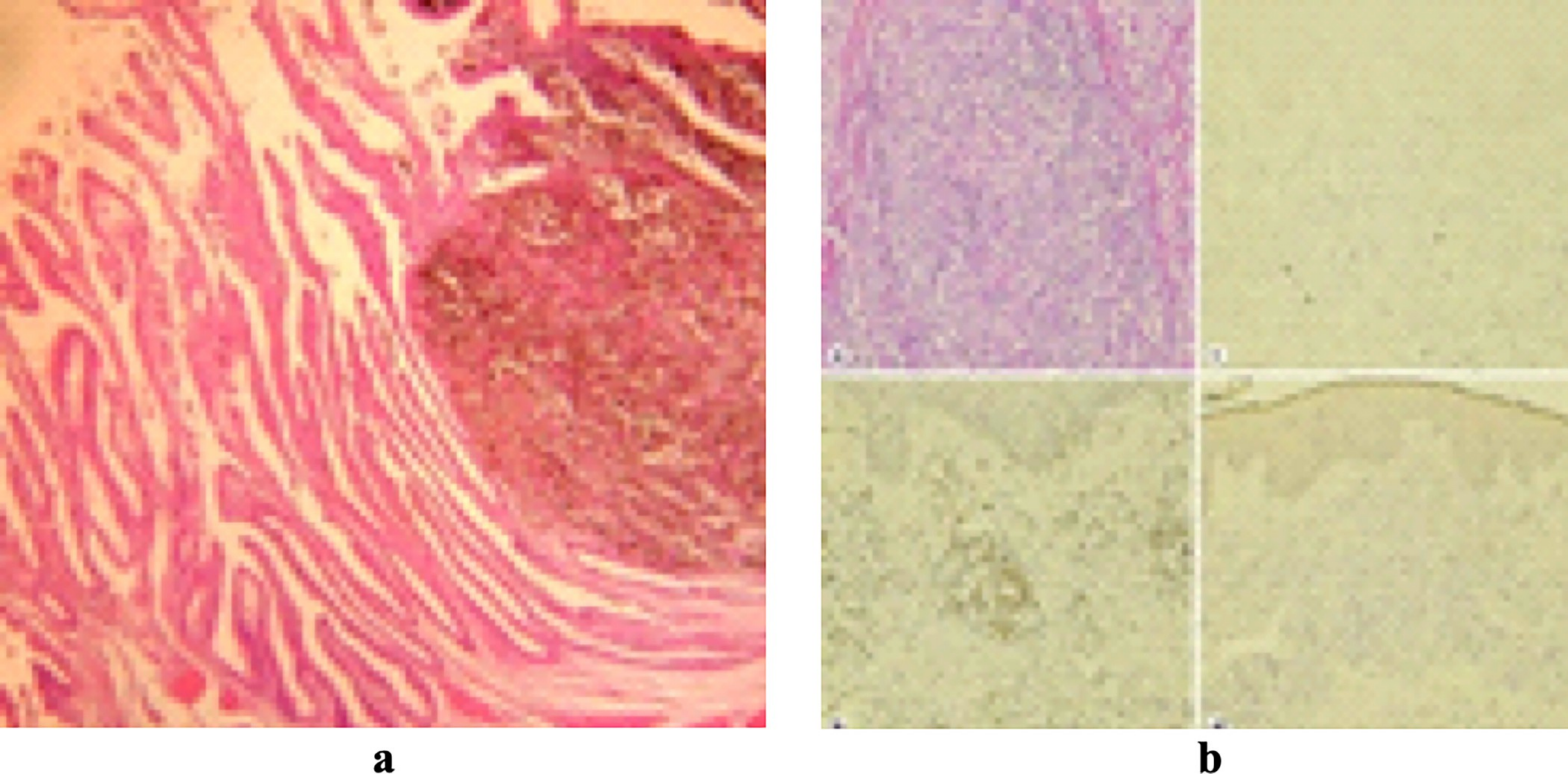
(a) Single-panel Image, (b) Multi-panel Image [2]. Reproduced with Permission from ImageCLEF 2013, and ImageCLEF2016.

Today, medical imaging is an important part of every stage of the healthcare process, from initial diagnosis to treatment selection and follow-up. Medical images have enormously increased over the recent past years [1]. The radiography studies only grew by 82% over the course of four years [2]. Each biomedical journal article typically comprises between 6.5 and 31 images on average [3]. These medical images appear in the form of single-panel images or multi-panel images. Single-panel images contain only one image, while multi-panel images, also called compound or composite images, are formed by combining multiple images to create one integrated image. Fig 1 depicts examples of both single-panel and multi-panel images.

The medical literature reports that about 50% of these images are multi-panel images [4, 5]‎. Multi-panel images have become an integral tool in medicine and research. For instance, a physician can easily represent a patient’s case by combining all of his imaging modalities such as X-rays, CT scans, and MRIs into one consolidated multi-panel image. Similarly, a researcher can easily represent the findings of his research experiments using multi-panel images. These increasing numbers of medical images require proper indexing to allow for fast and accurate retrieval from medical databases [6, 7].

**Fig 2:**
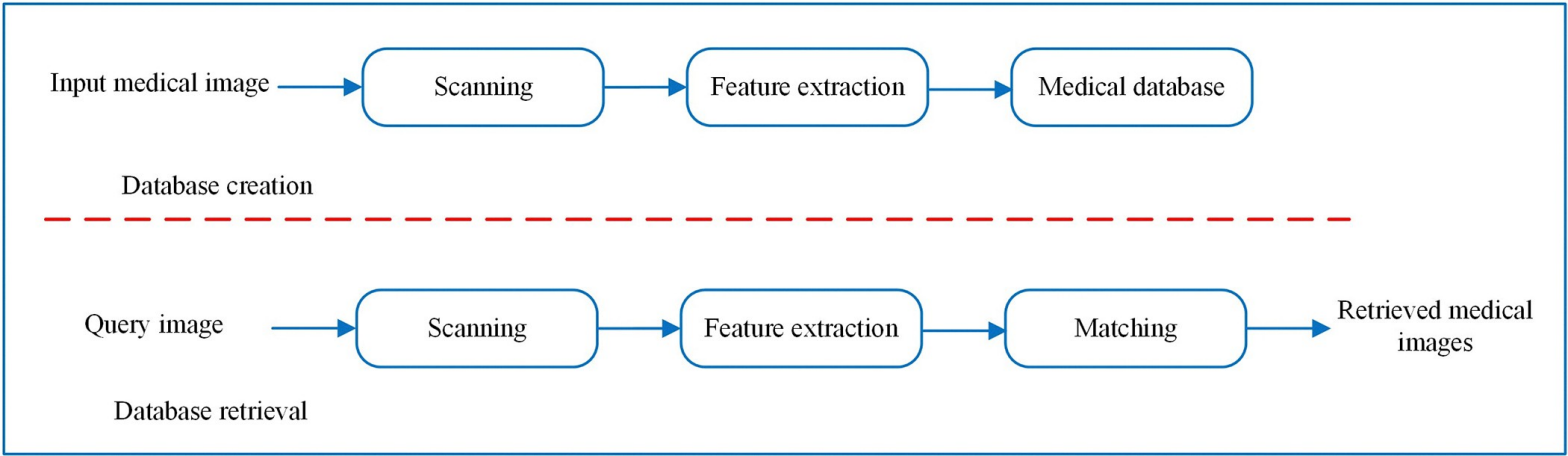
A Typical CBMIR system [4].

In content-based medical image retrieval (CBMIR) systems, images are stored and indexed according to their color, shape, and texture features [5–7]. When a user queries the system for an image, it extracts these features from the image and then scans the database in search of images that exhibit similar characteristics [8]. The system retrieves the relevant images matching these characteristics from the database. Fig 2 represents an overview of a typical CBMIR system.

Image retrieval systems have wide applications in various domains like medical, law enforcement, scientific, natural, sports, and entertainment [9, 10]. In medicine, image retrieval systems help doctors diagnose patients by finding similar medical images to compare with a given medical image. Similarly, image retrievals can be used in law enforcement in detecting criminals, and blocking offensive material on the web [11]. The archaeologists use image retrievals to retrieve digitized cultural heritage [12]. Image retrieval methods are effective for single-panel images, but face challenges with multi-panel images. This is because multi-panel images are considered single-panel images rendering their sub-images inaccessible [13–15]. To retrieve the sub-images, multi-panel images must be segmented into sub-images.

In the literature, several approaches are available for segmenting multi-panel images into sub-images. These approaches are observations-based and focus on identifying image borders and inter-panel separators to effectively separate sub-images in multi-panel images. These methods utilize image processing and statistical techniques to identify and locate image borders as well as sub-image separators. The method in [16] employs row and column sum computations to identify both horizontal and vertical borders. The method in [17] computes minimum projection to locate the positions of image borders and recursively segment the given multi-panel image in its sub-images. The method in [18] uses the statistical techniques of mean and variance, as well as the projection profiles to identify the borders locations in the multi-panel image. The approach in [19] computes the white space lines to detect the borders. The algorithms in [20] use a recursive approach to locate the borders using minimum projection profiles.

**Fig 3:**
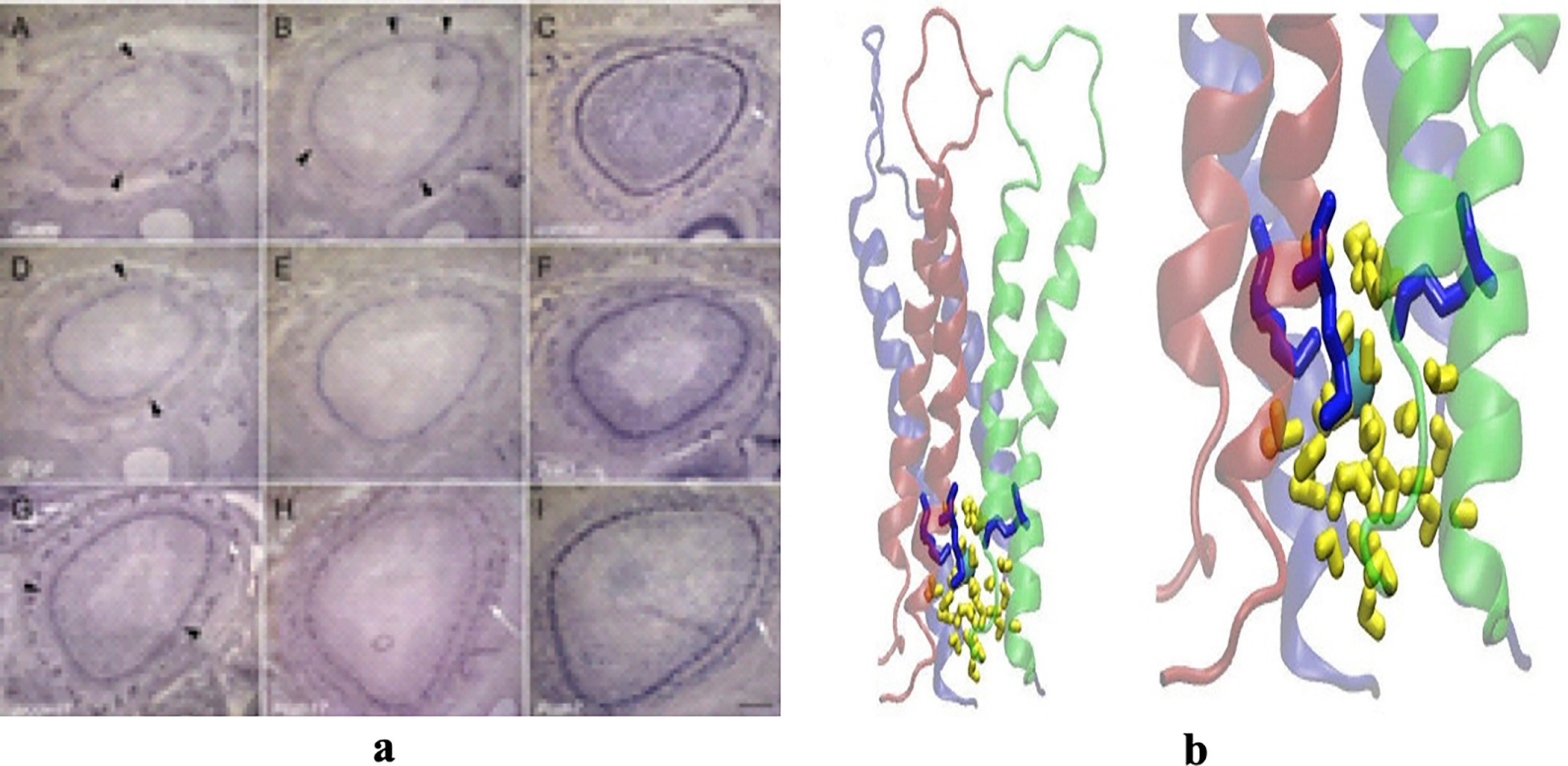
(a) Regular Multi-panel Image, (b) Irregular Multi-panel Image [15]. Reproduced with Permission from ImageCLEF 2016, and ImageCLEF 2014.

The approaches in ‎[21–23] perform well in segmenting regular multi-panel images i.e., the images with uniform space gaps or visible line separators between sub-images as shown in Fig 3(A).

However, these methods cannot segment irregular multi-panel images lacking visible gaps between panels [24]. The methods in [13, 19, 25] perform segmentations on irregular multi-panel images i.e., the images with non-uniform space gaps or invisible line separators between sub-images as illustrated in Fig 3(B).

These approaches also face segmentation issues posed by some specific images in the category of irregular multi-panel images. None of these methods can work to segment multi-panel images in the case of regular and irregular image types. To the best of the author’s understanding, no prior work has effectively addressed the segmentation problem of both types of multi-panel images.

In this study, we present a novel hybrid multi-panel image segmentation framework designed to segment regular as well as irregular images found in medical image datasets. The main contributions of our work are as follows.

1. We introduce an automatic image classification framework i.e. to identify the type of input image as single-panel image or multi-panel image, and then automatically categorizes the multi-panel image as either a regular or irregular multi-panel image.
2. Our method employs a hybrid approach for segmenting multi-panel images, including both regular and irregular multi-panel image types. Furthermore, it demonstrates a remarkable capability in accurately segmenting these multi-panel images in their component sub-images, thus improving the efficiency of content-based medical image retrieval systems.
3. Our approach utilizes the image projection profiles to identify the inter-panel borders in case of regular multi-panel images by computing row or column sums of the input image. For irregular multi-panel images, it employs basic image processing techniques using mathematical morphological operators such as connected components analysis. This allows our approach to be implemented on simple computers, enabling it a practical and cost-effective solution.
4. The proposed method analyzes multi-panel images and extracts information; a key step involves segmenting a multi-panel image in sub-images such that each one contains only a single image. This process aids in understanding the image and indexes these individual images in image database. A crucial step is enabling content-based image retrieval (CBIR) systems to effectively exploit the existing per image search practices of image search engines by retrieving the most relevant images for a query image.

Therefore, our framework demonstrates improved performance compared to the existing methods. Moreover, it can effectively segment a significant portion of the medical databases containing multi-panel medical images in sub-images, thus improving efficiency of the medical image retrieval systems.

The rest of the paper is organized as follows: First, the related work is reviewed. Next, the proposed framework is explained. The subsequent sections focus on the discussion of the experimental results and the details of the datasets used. The final section presents the conclusion and future work.

## 2. Related work

The segmentation of multi-panel images in sub-images is relatively unexplored research area, and only few papers address this challenge. The methods presented in these research works and their limitations highlight the need for alternative strategies to effectively address and explore this problem for its various applications [11, 12]. We provide an overview of these methods with their limitations.

The existing literature reports a number of methods for segmenting both regular multi-panel images and irregular multi-panel images [13, 19, 21–23, 25]. First, we review the segmentation methods available for regular multi-panel images as follows.

The regular segmentation methods are broadly categorized into the following two types: (1) content-based, and (2) content and caption-based approaches [21]. The content-based approaches for sub-image separation solely rely on using image content (i.e., pixel data) to detect the presence of gaps as sub-image separators and identify their locations [22, 26–29]. Most of the existing sub-image techniques are mainly based on finding gaps between sub-image panels. These gaps are solid (usually white or black) bands in a multi-panel image that are typically used to detect and separate sub-image panels. However, detecting gaps is challenge because of inconsistencies in image layout, especially in irregular images, where the gaps between sub-images are not very clear.

Alternatively, the methods using content and caption for separating sub-image panels rely on both the image content and caption to identify and segment multi-panel images in sub-images. In these methods, the image contents are used to process the image pixels’ data to search for the presence of sub-image separators [21–23]. The caption contents are used as a guide to identify the correct number of sub-image separations. However, it is impractical to use both image content and captions for sub-image separation because lables extraction is time-consuming and labor-intensive task.

The content-based approaches in [16, 17, 21] ‎use the image’s content (the pixel data) to detect the presence of gaps as sub-image separators and to identify the exact locations of these gaps. Once the locations of sub-images inside a given multi-panel image are determined, this information is used to slice and extract each sub-image from entire multi-panel image. The content-based segmentation methods are mostly observation-based such as (a) identifying image borders, and (b) identifying image inter-panel borders. The effectiveness of these methods relies on accurately identifying the borders and sub-image separators of the multi-panel image [21–23]. The existing literature reports the following content-based image segmentation techniques.

In [16], the method employs two recursive modules to find the locations of horizontal and vertical line separators in multi-panel images. First, the module utilizes the statistical measure of the image’s minimum projection to identify the locations of horizontal and vertical line separators along the sub-images. The next module identifies the locations of vertical separators within every horizontal segment after obtaining the horizontal sub-image segments. This approach is iteratively employed until the separation of all sub-images is achieved. The repeated detection of the same vertical line in each horizontal segment makes the method computationally expensive. The method cannot segment multi-panel images when there are no uniform space gaps between sub-images. The specific order of detecting horizontal separators first and then vertical separators also causes segmentation errors in some multi-panel images. Moreover, the method is not effective in segmenting multi-panel images with sub-images separated by non-straight lines or rectangular boxes.

In [17], the method uses the connected components analysis (CCA) technique to segment the multi-panel images. The technique involves identifying and labeling regions of connected pixels that share similar characteristics or belong to the same object. CCA can be preceded by thresholding to convert the image to binary form [30]. The process utilizes some connectedness criteria, like 4- or 8-connectivity, to group pixels. The accuracy of segmentation is confirmed by matching the image’s area with its sub-image summation. The proposed approach accurately separates sub-images in stitched images with high contrast. It, however, inaccurately separates the sub-images in low-contrast stitched images. Moreover, the method works only with stitched images, but a large portion of multi-panel images remains non-stitched.

In [21], the segmentation method uses horizontal and vertical projection profiles schemes to segment the multi-panel image. These projection profiles are the statistical measures of the rows sum (horizontal profile) or columns sum (vertical profile) of the image. Employing horizontal and vertical projection profiles methods, it computes the longest inter-panel borders in both horizontal and vertical directions. To detect the image’s longest inter-panel border, the method uses a dynamic threshold. To locate the lengthiest panel border along the horizontal direction and the lengthiest panel border along the vertical direction, the method analyzes the projection profiles by comparing them against the image’s width and height using a dynamic threshold. In case where the longest horizontal panel border is less than one-fourth of the image’s width, and the longest vertical panel border is also less than one-fourth of the image’s height, the image is classified as single-panel image. If not, the image will be multi-panel. Finally, in case where the longest horizontal border is longer than longest vertical one, the method will perform a horizontal segmentation first, otherwise it will carryout vertical segmentation. The method effectively segments the multi-panel images, but it fails to segment images with invisible sub-image separators or zigzag sub-images.

The method in [22] utilizes the projection profiles of the multi-panel image to determine the longest horizontal panel border alongside vertical panel border. Initially, the method pre-processes the image to determine whether it is single-panel or multi-panel through analysis of the lines separating sub-images. It segments the multi-panel image by using optimization techniques. The optimization approaches guide segmentation of the image with minimum number of sub-images. This reduces computational cost by comparing the detected number of lines in either horizontal or vertical directions. In cases where horizontal line counts are less than vertical line, the horizontal separation of the image is carried out first; otherwise, vertical separation occurs first. Finally, the dynamic programming optimization method is used in sub-image segmentation. The method segments the image with high segmentation accuracy and computing efficiency by using the dynamic approach. However, it faces performance issues if the image has invisible lines or has panels with irregular shapes and in case the images have broken lines between sub-images.

The method used in [23] employs the Hough transform to segment the multi-panel images with broken or disconnected lines between sub-images. The technique involves pre-processing the multi-panel image by transforming it into grayscale, followed by extracting horizontal alongside vertical edge images by applying an edge detection algorithm. The edge images with low contrast often contain broken lines causing inaccurate segmentation. These broken lines must be restored to get accurate results. To restore the disconnected lines, the method uses the Hough transform [31]. Initially, the approach identifies the locations of all boundary lines and sub-image panel lines. It employs a border identification model to accurately identify the actual boundary lines within the image [32]. The horizontal or vetical segmentation is performed by identifying the image’s horizontal or vertical lines. While this method generates accurate and optimized results using dynamic programming techniques, it fails in segmenting multi-panel images with irregular sub-images or invisible inter-panel image’s borders.

Next, we explore the approaches in the available literature for segmenting irregular multi-panel images as follows.

The literature reports different approaches for the segmentation of irregular multi-panel images [13, 19, 25]. These methods work by identifying connected contents inside each panel of a multi-panel image.

In [13], the approach begins by identifying connected contents within each panel and utilizing connected components analysis (CCA) for detection of indidual panels to separate these panels into their constituent sub-images. However, the technique comes across challenges with over-segmentation, where isolated small objects are misinterpreted separate panels and are segmented away from the primary image panel.

In [19], the method employs a connected components analysis (CCA) apporach for segmenting a multi-panel image in sub-images. The multi-panel image is pre-processed to make the gaps between sub-images more visible for segmentation. The method includes an additional step i.e. the quality assessment phase of segmentation is implemented to ensure the accuracy of the segmentation process, specifically ensuring that only the appropriate number of sub-images inside multi-panel image are correctly identified and recovered. It accurately segments multi-panel images, but the segmentation quality step makes it computationally expensive. Moreover, it fails to segment multi-panel images where both the image panels and gaps vary in size, or the images with no uniform space gaps which cause under- and over-segmentation issues.

In [25], the proposed method also deals with separating sub-images in stitched compound images by applying the connected component analysis technique. The approach overcomes the over- and under-segmentation issues through a quality assessment step. In the quality assessment step, panel overlapping objects are combined, connected components with small regions are removed, and omitted panels are recaptured by examining the free space close to the detected panels. The segmentation accuracy is verified by matching the overall multi-panel image’s area with sum of its all sub-images’ areas. The proposed method generates accurate results in separating sub-images in stitched images with significant contrasts. However, it inaccurately separates the sub-images in low-contrast stitched images. Moreover, it is computationally expensive due to the quality assessment process. Additionally, it only works with stitched images, but most multi-panel images are non-stitched. Furthermore, it faces over-segmentation and under-segmenting issues in blurred, fragmented, and stitched multi-panel images.

The review of existing literature reveals no comprehensive framework for classifying and segmenting both regular and irregular multi-panel images [21, 22, 33, 34]. This paper proposes a novel hybrid framework designed to effectively segment these images in medical datasets.

### 2.1. Hybrid multi-panel image segmentation framework

**Fig 4:**
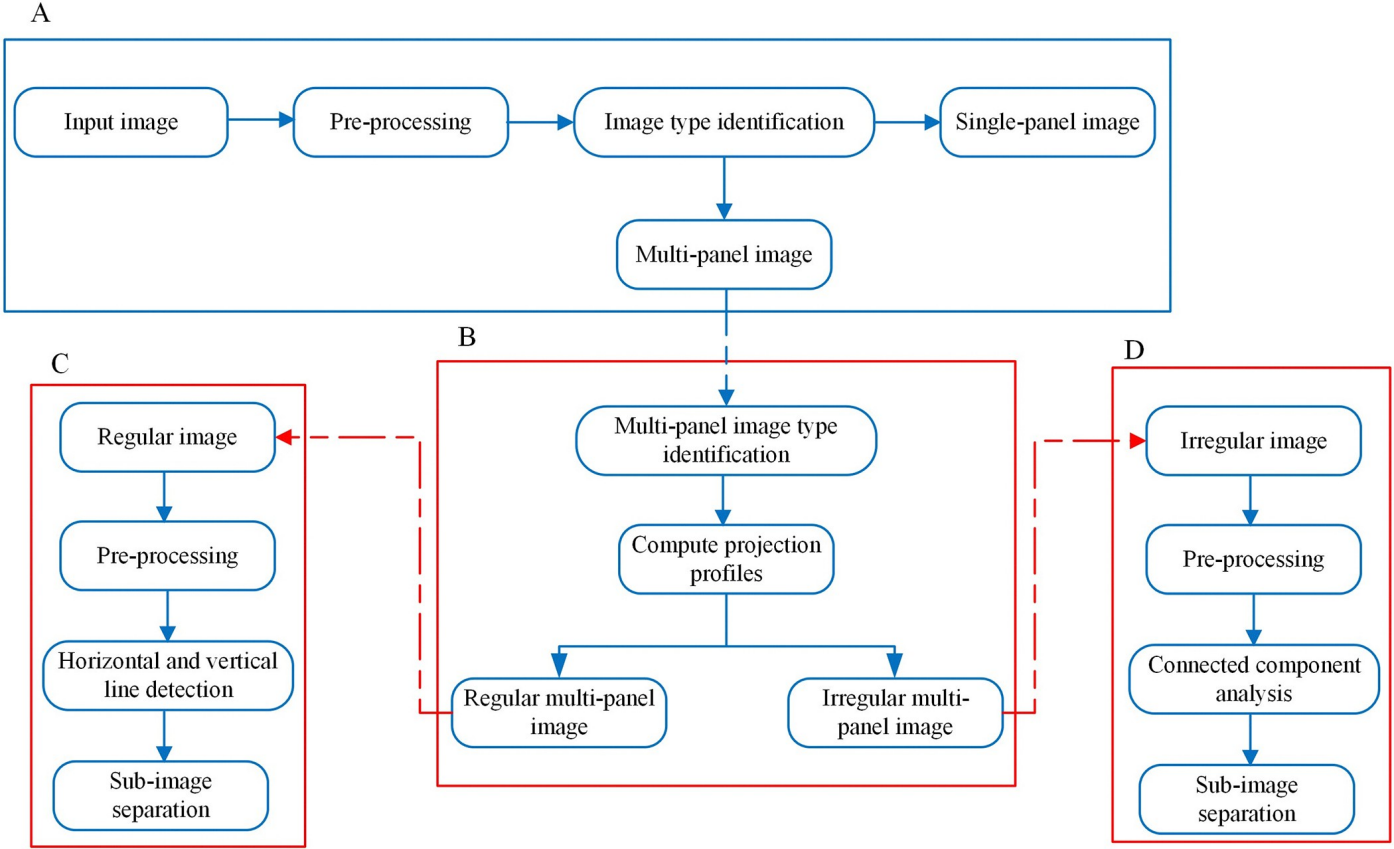
(A) Image Type Identification, (B) Multi-panel image Type Identification, (C) Regular Multi-panel Image Segmentation, (D) Irregular Multi-panel Image Segmentation.

This section outlines the framework being proposed, comprising four essential modules illustrated in Fig 4. Following is a detailed explanation of each module.

### 2.2. Image type identification

**Fig 5:**
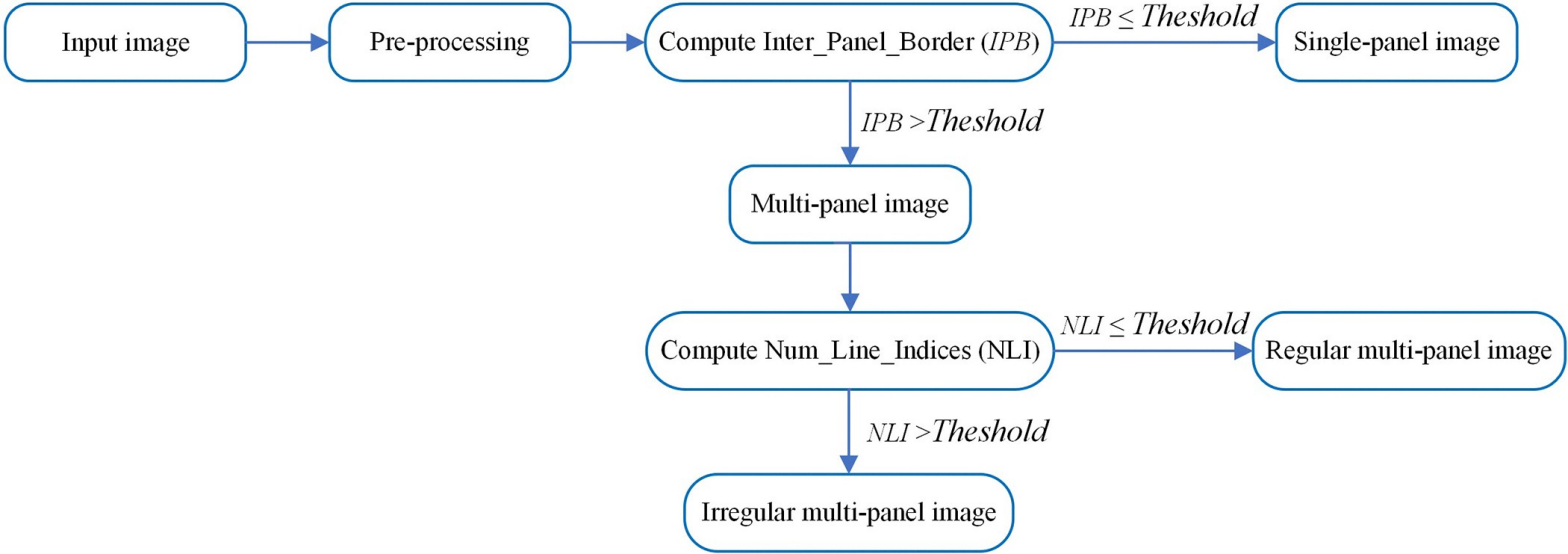
Flowchart of image type iIdentification.

In this module, the system determines whether the input image is a single-panel or multi-panel image. Furthermore, the multi-panel image is categorized as either regular or irregular multi-panel image. The flowchart illustrated in Fig 5 represents the image identification process.

To determine type of the image, the framework initially receives the image in RGB color and then transforms it into grayscale image. For each image, horizontal and vertical projection profiles are computed based on the sum of pixels in each row and column, respectively. These projection profiles are then examined to find the locations and the total number of the horizontal and vertical panel borders within the boundary of the multi-panel image. When the count of inter-panel borders falls below a specified threshold, the framework classifies the image as a single-panel image. If the count exceeds this threshold, the system identifies the image as multi-panel. The multi-panel image is further categorized as either regular or irregular multi-panel image based on the total number of inter-panel borders, using a predefined threshold. Algorithm 1 outlines the type identification process for the input image as follows.

**Algorithm 1 Image Type Identification**

**Input:** *Image im*, *Integer threshold_single*, *Integer threshold_comp*

**Output:** *single-panel or multi-panel image*

1 *Igray (x*, *y)* ← *im*

2 *HLV(i)* ← $\sum_{j=1}^{N}Igray(i,j)$

3 *VLV(j)* ← $\sum_{i=1}^{M}Igray(i,j)$

4 **if** *(HLV < threshold_single and VLV < threshold_single*) **then**

5 *single-panel* ← *im*

6 return *single-panel*

7 **else** *multi-panel* ← *im*

8 **end if**

9 return *multi-panel*

Where *HLV* and *VLV* represent the horizontal and vertical line vectors, respectively, while *M* and *N* indicate the input image’s height and width. *Threshold_single* and *threshold_comp* denote the thresholds for single-panel and multi-panel images, respectively. The framework determines if an image is single-panel or multi-panel by analyzing the horizontal line vector (*HLV*) and vertical line vector (*VLV*). These vectors are derived from horizontal and vertical projection profiles, as defined in Eq (1) and Eq (2), respectively

The variables *h*l(1), *h*l(2), …, *h*l(*n*) represent the indices of horizontal lines, while *v*l(1), *v*1(2), …, *v*l(*m*) indicate the indices of vertical lines in the given image.

Once these vectors are obtained, their contents are analyzed to decide the image’s type. An image is classified as single-panel if the contents of both vectors *HLV* and *VLV* are empty, otherwise, it is declared as multi-panel image, where a threshold of 0 represents single-panel image and any value above 0 indicates a multi-panel image.


HLV=[hl(1),hl(2),…,hl(n)] (1)

VLV=[vl(1),vl(2),…,vl(m)] (2)

### 2.3. Multi-panel image type identification

The procedure of type identification of the multi-panel image is described by Algorithm 2 as follows.

**Algorithm 2 Multi-panel Image Type Identification**

**Input:** *Image multi-panel*, *Integer HLV[]*, *Integer VLV[]*, *Integer threshold_comp*

**Output:** *regular_multi-panel or irregular_multi-panel image*

1 *TotalHoriontalLines* ← FindTotalLines(*HLV*)

2 *TotalVerticalLines* ← FindTotalLines (*VLV*)

3 **if** *(TotalHoriontalLines < = threshold_comp* **or** *TotalHoriontalLines < = threshold_comp)* **then**

4  *regular_multi-panel* ← *multi-panel*

5  return *regular_multi-panel*

6 **else if** *(TotalHoriontalLines > threshold_comp* **or** *TotalHoriontalLines > threshold_comp)* **then**

7  *irregular_multi-panel* ← *multi-panel*

8 **end if**

9 return *irregular_multi-panel*

**Fig 6:**
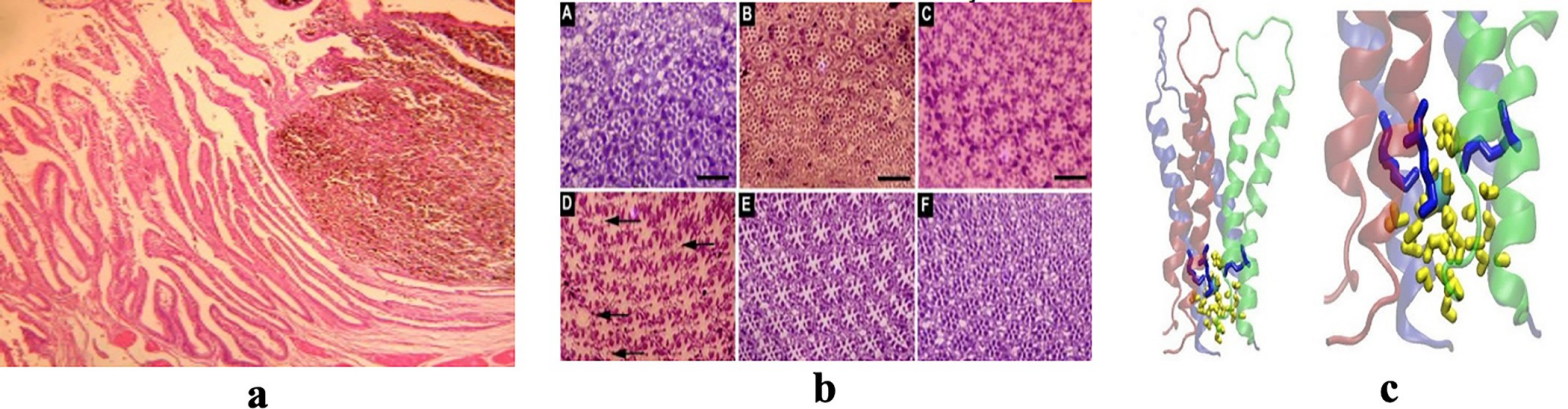
Results of image type identification algorithm. (a) Single-panel Image, (b) Regular Multi-panel Image, (c) Irregular Multi-panel image. Reproduced with Permissions from ImageCLEF 2013, ImageCLEF 2016, and ImageCLEF 2014.

Where the variables *TotalHoriontalLines* and *TotalVerticalLines* represent the horizontal line and vertical lines indices, computed from *HLV* and *VLV*, respectively. The threshold *threshold_comp* shows the threshold value for a multi-panel image. The vectors are further examined to make decisions about multi-panel image types. A multi-panel image is considered a regular multi-panel image or irregular multi-panel image based on a pre-defined threshold on the contents of *TotalHoriontalLines* and *TotalVerticalLines* vectors. The image is a regular multi-panel image if there are fewer line indices than the defined threshold, otherwise, it is declared irregular multi-panel image. (i.e., a threshold value defined as in the range of 1…5 will represent the regular multi-panel image, and the contents of line indices above this threshold will represent the irregular multi-panel image). All these defined thresholds are observation-based and have been tested on input images with satisfactory results. For single-panel image cases, the threshold 0 represents the absence of inter-panel borders which defines single-panel images while a threshold value more than 0 indicates the presence of panel borders in the image which defines a multi-panel image. In multi-panel images, the maximum observed number of inter-panel borders separating the sub-images, whether horizontally or vertically, is five. Based on these observations, our framework can successfully categorize the input image into one of three types: single-panel image, or multi-panel image and multi-panel image with further classification into either regular or irregular image. For instance, the *HLV* and *VLV* values for the images in Fig 6 are derived using Eq (1) and Eq (2) as follows. For the image in Fig 6(A): *HLV* = [], and *VLV* = []. Regarding the image in Fig 6(B): *HLV* = [119], *VLV* = [153, 309]. For the image in Fig 6(C), *HLV* = [1–8, 298–311], and *VLV* = [1–40,181–266, 559–599]. Let’s examine the outcomes of image type identification algorithm when applied to these images. For the image Fig 6(A), the image’s horizontal line indices (*HLV*) and vertical line indices (*VLV*) are both represented as empty sets. This suggests the image is a single-panel one, with no sub-images or panels. Next, for the image in Fig 6(B), *HLV* is represented as [119], and *VLV* is shown as [153, 309]. These indices indicate that the image is a regular multi-panel image, where the panels are systematically organized either horizontally or vertically. Finally, in Fig 6(C), the horizontal line indices spans over [1–8, 298–311], while the vertical line indices spread over [1–40, 181–266, 559–599]. These range-based indexing patterns provide clear indications that the image belongs to the category of irregular multi-panel images, where the panels exhibit a more diverse and non-uniform arrangement. Using this data, the identified line indices provide valuable information about the particular type of image.

### 2.4. Regular multi-panel image segmentation

**Fig 7:**
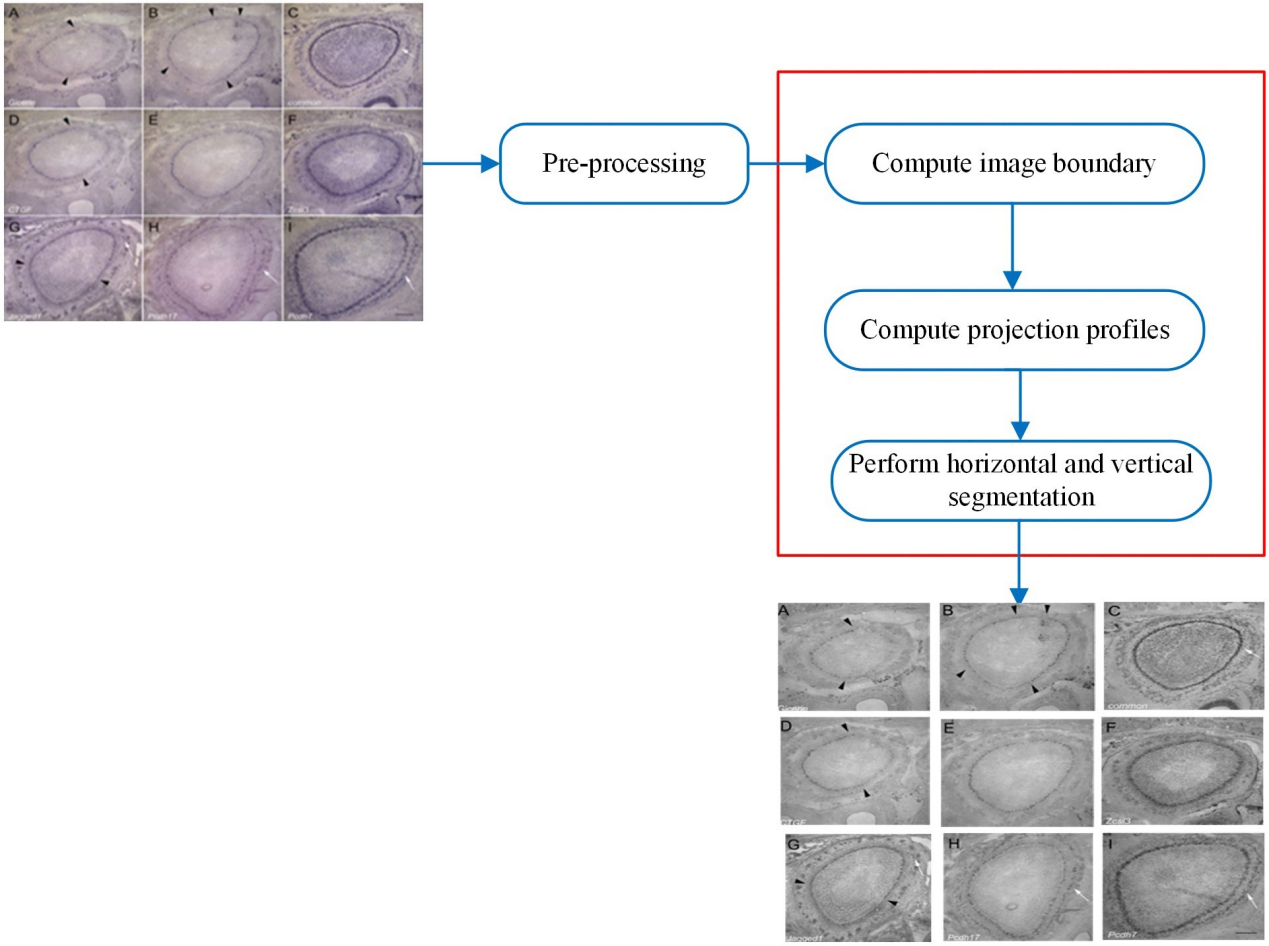
Regular multi-panel image segmentation. Reproduced with Permission from ImageCLEF 2016.

This section describes the segmentation of regular multi-panel images. Fig 7 shows our proposed framework. The details of the various modules with the relevant algorithms are described as follows.

### 2.5. Pre-processing

This module converts the color input image to grayscale as follows.

Grayscale=0.2989*Red+0.5870*Green+0.1140*Blue (3)

Eq (3) computes the grayscale value from the color input image, where "Red", "Green" and "Blue" represent the individual color channel values of a pixel in the RGB image in the range from 0 to 255. The coefficients (0.2989, 0.5870, and 0.1140) represent the human perception of colors. They are used to calculate the weighted average of the color channels to determine the pixel’s grayscale intensity. Applying this formula to each pixel in the RGB image gives the corresponding grayscale intensity value, which ranges from 0 (black) to 255 (white). This creates a grayscale image *Graysclae* where each pixel represents the brightness of the original color pixel. After pre-processing, the image moves to the following module, where it removes the outer border of the image.

#### 2.5.1. Image border identification

The border of the input image, *im*, is computed using Eq (4) as follows.

image_border=im[top_rows:bottom_rows,left_columns:right_columns] (4)

This resultant image with no image boundary is moved to the following module, where it computes the horizontal and vertical image histograms.

#### 2.5.2. Compute horizontal image histogram

We use the Eq (5) to compute the horizontal histogram for each row *i* by summing up the intensities along that row as follows.

$$H(i)=\sum_{i=1}^{W}\;im[i,j]$$ (5)

Where *H*(*i*) represents the horizontal histogram value for row *i*, *W* indicates the image’s width, while *im*[*i*,*j*] shows the intensity value at row *i* and at column *j*.

#### 2.5.3. Compute vertical image histogram

The vertical histogram *V(j*) for each column *j* is computed as the sum of pixel intensities along that column using Eq(6).

$$V(j)=\sum_{j=1}^{H}\;im[i,j]$$ (6)

Where *V*(*j*) is the vertical histogram value for column *j*, *H* denotes the image’s height (number of rows), while *img*[*i*,*j*] indicates the intensity value at row *i* and column *j*.

The horizontal histogram value *H(i)* and vertical histogram *V(j)*are used to compute the high intensity rows and high intensity columns.

#### 2.5.4. Compute projection profiles using high intensity rows

To compute high-intensity rows on threshold *T* using the horizontal histogram *H(i)* for each row *i* of an image *im*, Eq (7) is used as follows.

HighintestiyRows={i|H(i)>T} (7)

Where *T* is the assumed intensity threshold, the set of high-intensity rows, *HighIntensityRows*, consists of all rows (*i*) for which the horizontal histogram value *H(i)* is greater than the specified threshold, *T*.

#### 2.5.5. Compute projection profiles using high intensity columns

To compute high-intensity columns on threshold *T* using the vertical histogram *V*(*j*) for each column *j* of an image *im*, Eq (8) is used as follows.

HighintestiyColumns={j|V(j)>T} (8)

Where *T* is the assumed intensity threshold, the set of high-intensity columns, *HighIntensityColumns*, consists of all columns (*j*) for which the vertical histogram value *V(j)*) is greater than the specified threshold, *T*.

**Fig 8:**
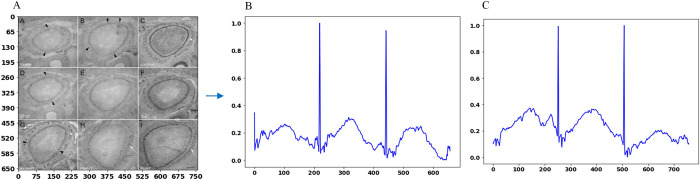
(A) Input Image, (B) Horizontal Projection Profiles, (C) Vertical Projection Profiles.

The horizontal and vertical projection profiles are averaged to create mean projections, which preserve more image information by dividing each row or column’s pixel intensity sum by the image’s width and height, respectively. The horizontal and vertical projection profiles of the input image are illustrated in Fig 8.

### 2.6. Perform horizontal segmentation

In this module, the multi-panel image is segmented into individual sub-images [35]. To determine whether to follow horizontal or vertical segmentation first, we analyze the contents of two lists: horizontal line vector(*HLV*) and vertical line vector(*VLV*). If the number of elements in *HLV* exceeds that of the *VLV*, we proceed with horizontal segmentation; otherwise, vertical segmentation is chosen. Algorithm 3 outlines the steps of horizontal segmentation.

**Algorithm 3 Horizontal Segmentation**

**Input:** *Image img*, *Integer high_intensity_rows []*, *Integer start_row*, *Integer end_row*

**Output:** Horizontally segmented sub-images *images[]*

 1 *high_intensity_rows* ← *[r1*, *r2*, *…*, *rk]*

2 *columns* ← FindTotalColumns*(img)*

3 *rows* ← FindTotalRows(*img*)

4 *images* ← [ ]

5 *start*_row ← 0

6 **for each** *end****_****row* **in** *high_intensity_rows* **do**

7 h_img ← crop_img (*img*, *start*_*row*, *end_row*, 0, *columns*)

8 *r*, *c* ← shape(*h_img*)

9 hb_img ← compute_image_boundary (h_img, 1, r−1, 1, c−1)

10 images. append (*hb_img*)

11 *start_row* ← *end_row*

12 **end for**

13 *images*. append (crop_img (*img*, *start_row*, *rows*,0, *columns*))

14 return images

Where, the method ‘*crop_img* (*img*, *start*_*row*, *end*_*row*, 0, *columns*)’ represents the operation of cropping the image from *start*_*row* to *end*_*row* and from *column* 0 to *columns*. The method ‘*compute*_*image*_*boundary* (*h_img*,1, *r−1*,1, *c−1*)’ represents the operation of computing the image boundary for the segment *h_img* from rows 1 to *r−1* and columns 1 to *c−1*. The list ‘*images’* are then updated with the segmented images.

### 2.7. Perform vertical segmentation

The steps in algorithm 4 are followed to perform vertical segmentation. It iterates through the specified *high_intensity_columns*, extracts vertical segments using the *crop_img* method, computes boundaries using the *compute*_*image*_*boundary* function, and appends the segmented images to the *images* list. Finally, it adds the last segment after the loop to cover the remaining part of the image.

**Algorithm 4 Vertical Segmentation**

**Input:** *Image img*, *Integer high_intensity_columns []*, *Integer start_row*, *Integer end_row*

**Output:** Vertically segmented sub-images *images[]*

  1 *high_intensity_columns* ← [*c1*, *c2*,…, *ck*]

2 *columns* ← FindTotalColumns*(img)*

3 *rows* ← FindTotalRows(*img*)

4 *images* ← [ ]

5 *start*_column ← 0

6 **for each** *end****_****column* **in** *high_intensity_columns* **do**

7 v_img ← crop_img (*img*, 0, *rows*, *start_column*, *end_column*)

8 *r*, *c* ← shape(*v_img*)

  9 *vb_img*  ←  compute_image_boundary (*v_img*, 1, *r-1*, 1, *c-1*)

10 *start_column* ← *end_column*

11 images. append (vb_img)

  12 **end for**

13 *images*. append (crop_img (*img*, 0, *rows*, *start_column*, *columns*))

14 return *images*

Where, the method ‘*crop*_*img* (*img*, *0*, *rows*, *start_column*, *end_column*)’ represents the operation of cropping the image from row *0* to *rows* and from *start_column* to *end_column*. The method ‘compute_image_boundary (*v_img*,*1*, *r−1*,*1*, *c−1*)’ represents the operation of computing the image boundary for the segment *v_img*. The list ‘*images’* are then updated with the segmented images.

### 2.8. Irregular multi-panel image segmentation

**Fig 9:**
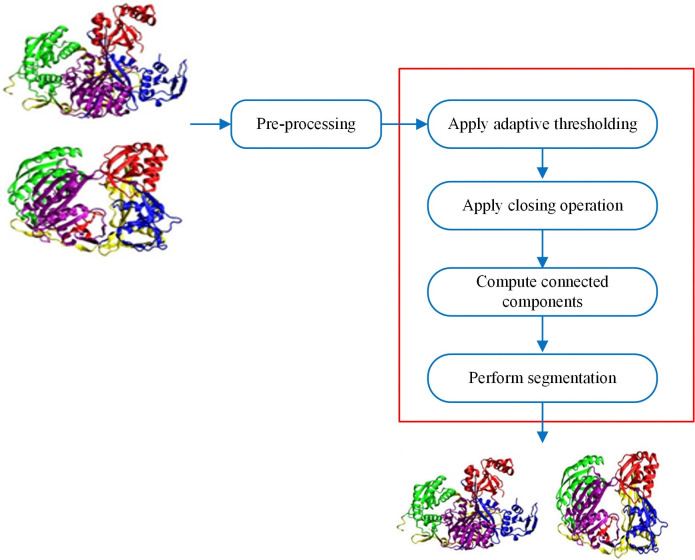
Overview of the irregular multi-panel image segmentation. Reproduced with Permission from ImageCLEF 2015.

The proposed irregular multi-panel image segmentation framework consists of the following five main modules, performing pre-processing, adaptive thresholding, closing morphological operation, computing connected components, and performing segmentation if the connected components satisfy the defined threshold criteria. Fig 9 provides an overview of our multi-panel image segmentation framework.

The details of each module with an explanation of the algorithm used are discussed as follows.

### 2.9. Pre-processing

This module aims to transform an input color image to a grayscale image using Eq 3). This grayscale image is moved to the next module to perform adaptive thresholding on it and to highlight regions of interest accurately.

#### 2.9.1. Apply adaptive thresholding

This module is used to apply adaptive thresholding on the grayscale image. The algorithm adaptively separates objects from the background in the grayscale image using a threshold value calculated based on Otsu’s method. This is particularly useful when lighting conditions vary across the image, as it helps to accurately highlight regions of interest. Algorithm 5 implements the adaptive threshold used in this module on grayscale images as follows.

**Algorithm 5 Perform Adaptive Thresholding**

**Input:** *Image grayscale*, *Integer thresholdValue*, *Integer maxValue*

**Output:**  Thresholded image *Thresh*

  1 *thresholdValue*  ← calculateOtsu’sThresholdValue(*grayscale*)

2 **for all** *pixels(x*, *y*) ϵ *grayscale* **do**

3 **If** (*pixel(x*, *y)* < = *thresholdValue*) **then**

4 *Thresh(x*, *y)* ←  0

5 **else if** *(pixel(x*, *y) = = maxValue)* **then**

6 *Thresh(x*, *y*)  ←  maxValue

7 **end if**

8 **end for**

  9 return *Thresh*

where, the variables *grayscale*, *thresholdValue*, and *maxValue* represent the grayscale image, the threshold value calculated by Otsu’s method, and the maximum value (which is typically 255 in a ograyscale image), respectively. The algorithm performs threshold analysis on grayscale images using Otsu’s method [36]. It automatically calculates the optimal threshold based on intensity to separate objects from the background. Pixels below the threshold are part of the background (set to 0), while pixels greater than the threshold are part of the foreground (set to the specified maximum value). This highlights interesting objects in the image.

#### 2.9.2. Apply closing morphological operation

In this phase, the "closing" morphological operation is performed on the binary image (thresholded image). The "closing" morphological operation removes small holes or gaps in regions of interest in the binary image. This is achieved through dilation followed by erosion, which fills in gaps and smooths object edges. This operation is particularly useful in image processing tasks such as reducing noise and enhancing the shape of objects. Algorithm 6 implements the morphological operation “closing” on the thresholded image as follows.

**Algorithm 6 Perform Morphological Closing Operation**

**Input:** *Image Thresh*

**Output:**  Image  *Closing*

  1 *kernel*   ←  createKernel(*k_size1*, *k_size2*)

2 **for all** *pixels*(x, y) ϵ *Thresh* **do**

3 *neighborhood*_t ←  extractNeighborhood(*Thresh*, ke*r*nel)

4 *Dilated(x*, *y)*  ←  getMaximumValue(*neighborhood_t*)

5 **end for**

6 **for all** *pixels(x*, *y) ϵ Dilated* ***do***

7 *neighborhood_d*  ←  extractNeighborhood(*Dilated*, *kernel*)

8 *Closing(x*, *y)* ←  getMinimumValue(*neighborhood_d*)

9 **end for**

10 return *Closing*

Where the variable *Thresh* represents the threshold binary image. The algorithm creates a small kernel with dimensions *k_size1* and *k_size2* (typically 29x29). It then uses this kernel to extract the surrounding neighborhood of each pixel within the thresholded image. Next, the dilation operation is performed by finding maximum value in the extracted neighborhood. Lastly, the closing operation is performed by finding the minimum value within the extracted neighborhood.

#### 2.9.3. Compute connected components

This phase aims to calculate the connected regions in an image that has been processed with the “close” operation. These connected regions, called connected components, are distinct regions of connected pixels within a binary image that are commonly used for object detection, analysis, and labeling. It labels these components and extracts key statistics such as location, dimensions, and area of each labeled area. This information is critical for tasks such as identifying, tracking, and analyzing objects in image processing applications. Algorithm 7 implements the procedure to compute the connected components in a closed image as follows.

**Algorithm 7 Compute Connected Components**

**Input:** *Image Cloisng*

**Output:**  Connected components with the positions *x*, *y*,  the dimensions *w*, *h*, and area *Area*

  1 *(numLabels*, *labels*, *stats*, *centroids) ←* connectedComponentsWithStats*(Closing)*

2 **for all** *i ϵ (1*.. *numLabels)*  **do**

3 *x* ← *stats(i)(Left)*

4 *y* ← *stats(i)(Top)*

5 *w* ← *stats(i)(Width)*

6 *h* ←  *stats(i)(Height)*

7 *Area* ←  *stats(i)(Area)*

8 **end for**

Where the variable *Closing* represents the closed image. The algorithm collects statistics about the connected components in the closed image, like their positions (*x*, *y*), dimensions (*width* and *height*), and *Area*. This statistical information is used in object identification and analysis.

### 2.10. Perform segmentation

The objective of this algorithm is to perform segmentation on an image that has been processed to identify the connected components and compute their statistics. It extracts the region of interest in the image by considering the size of region of interest in the image. It achieves this by extracting rectangular regions from the image based on a defined region threshold. This can be useful to highlight significant parts of an image and exclude smaller or less relevant areas. Algorithm 8 implements the segmentation process as follows.

**Algorithm 8 Segmentation**

**Input:** *Image im*, *Integer numLabels*, *Integer x*, *y*, *w*, *h*, *Area*, *Area_threshold*

**Output:** *Image segments*

  1 **for all** *i ϵ (1*.. *numLabels)* **do**

2 **If** (*Area > Area_threshold*) **then**

3 *segmented_area* ← extractRectangularRegion**(***i****)***(*img*, *x*, *y*, *w*, *h*)

4 **end if**

5 **end for**

where, the variables *image*, *numLabels*, *x*, *y*, *w*, *and h* denote the closed image, total number of labels, the pixel positions (*x*, *y*), dimensions (*width* and *height*), and *Area* computed in the previous module. The algorithm segments the image using these statistical parameters and a predefined area *Area_threshold*. It is our observation that the choice of threshold affects the segmentation results significantly. The empirically selected *Area_threshold* produced better segmentation results based on human verification.

## 3. Experimental results and discussion

This section evalutes the proposed framework’s experimental results on our test dataset. The framework classifies medical images into one of three categories: single-panel image, regular multi-panel or irregular multi-panel image. Additionally, the framework includes a segmentation phase for multi-panel medical images. The evaluation comprises several stages, starting with the initial categorization of images as single-panel or multi-panel, followed by additional classification for multi-panel images into regular multi-panel images (i.e., with clear horizontal or vertical panel separation) or irregular multi-panel images (i.e., with no horizontal or vertical separation), and no processing is performed in the case of single-panel images. Segmentation of regular or irregular multi-panel images into sub-images is performed by applying the corresponding segmentation module for regular multi-panel images and irregular multi-panel images. Our framework’s performance is evaluated on a medical dataset containing medical images.

### 3.1. Datasets

The framework is evaluated on a collection of 5750 images selected from the publicly available datasets [14, 15]: ImageCLEFmed 2013, ImageCLEFmed 2014, ImageCLEFmed 2015, and ImageCLEFmed 2016. To evaluate the performance of our hybrid framework on both regualr and irregular multi-panel images, we compare our results with the reporeted works in the literature for regular images [21–23] and for irregular multi-panel images [13, 19, 25].

In this dataset collection, there are 500 single-panel images and 5250 multi-panel images. Multi-panel images are categorized into two types: i) regular multi-panel images and ii) irregular multi-panel images. The first group consists of 3120 multi-panel images and the second group consists of 2130 multi-panel images. This dataset is divided into training and testing datasets, where the training dataset comprises 370 single-panel images and 70% of the multi-panel images from each category (2184 regular, 1491 irregular), whereas the testing dataset includes 130 single-panel images and the remaining 30% of the multi-panel images from each category (936 regular, 639 irregular).

### 3.2. Performance evaluation

The evaluation was conducted using precision, recall, and F1-score parameters to evaluate the performance of our framework [37–41]. These parameters are evaluated using Eqs (9), (10), and (11) as follows:

$$Precision=\frac{Nc}{Ne}$$ (9)

$$Recall=\frac{Nc}{Nt}$$ (10)

$$F1-score=\frac{2\times Precision\times Recall}{Precision+Recall}$$ (11)

Where, *Nc*, *Ne*, *Nt* represent the number of correctly identified sub-images, total number of extracted sub-images, and the ground truth (total number of sub-images), respectively. Our approach follows a manual verification process to assess the accuracy of the outcomes produced by the image type identification module. The module’s average accuracy is obtained when the number of correctly identified single-panel images is divided by the total number of single-panel images in the test dataset. The same approach is used to calculate the module’s average accuracy for multi-panel images in the test dataset. Following the evaluation methodology outlined for the image’s type identification module, a consistent manual procedure is used to assess the accuracy of sub-images extracted from multi-panel images. Sub-images are recognized as correct when the overlap area between sub-images and ground truth sub-images exceeds two-thirds (66%) of the ground truth sub-image’s area; otherwise, they are assumed as incorrect. Next, to assess the performance of the proposed framework compared to existing methods reported in the literature, the average precision, recall and F1score are evaluated on the entire test dataset [42–47].

#### 3.2.1. Performance comparison

The results of our proposed framework are evaluated against existing methods for segmenting both regular and irregular multi-panel images [13, 19, 21–23, 25]. The performance of these methods is measured using the metrics specified by Eqs (9), (10), and (11) in the performance evaluation section.

### 3.3. Comparison with regular image segmentation methods

In this section, the accuracy of results of the framework is compared with existing regular multi-panel image segmentation methods in [21–23] as follows. Table 1 reports that the proposed method shows the potential to automatically classify input images into their respective types. It performs this task with remarkable efficiency, taking 94 seconds to identify a single-panel image and 94.50 seconds to identify a regular multi-panel image or irregular multi-panel image. The results demonstrate that our method outperforms existing methods in terms of CPU time for image type identification.

**Table 1:** Performance comparison of our method with existing methods in image type identification.

Methods	Image type identification (in seconds)	
	Single panel	Multi-panel (regular and irregular images)
Framework [21]	95.78	95.05
Dynamic Programming [22]	95.78	95.05
Method [23]	95.78	95.05
Proposed method	**94.0**	**94.50**

Table 2 highlights the results’ accuracy of our approach with existing approaches during input image type identification phase. The methods [21–23] do not consider the irregular image type identification and are indicated by “Nil”. ’ in the table. The proposed approach can identify both regular and irregular multi-panel images with a significant accuracy of 96.10%. This highlights the improved performance of our framework compared to existing methods in multi-panel image type identification.

**Table 2:** Comparison based on sub-image segmentation accuracy in terms of percentage.

Methods	Multi-panel image type identification (in percentage)	
	Regular multi-panel	Irregular multi-panel
Framework [21]	95.05	Nil
Dynamic Programming [22]	95.05	Nil
Method [23]	95.05	Nil
Proposed method	**96.10**	**96.10**

Table 3 displays a comparison of the accuracy of our approach against other methods in segmenting multi-panel images in their sub-images. The methods in [21–23] have accuracy with an average precision value as 96.29, an average recall value of 89.40, and an average F1-measure of 92.72, respectively during multi-panel image segmentation. In contrast, the proposed method performs the multi-panel image segmentation task with improved accuracy of 97.10 for average precision, 97.10 for average recall, and 97.10 for F1-measure. The results indicate that our framework is effective at segmenting sub-image panels within multi-panel images compared to other methods.

**Table 3:** Comparison based on sub-images segmentation accuracy in terms of percentage.

Methods	Sub-images segmentation(in percentage)		
	Average precision	Average recall	Average F1-measure
Framework [21]	96.29	89.40	92.72
Dynamic Programming [22]	96.29	89.40	92.72
Method [23]	96.29	89.40	92.72
Proposed method	**97.10**	**97.10**	**97.10**

Table 4 illustrates the processing times of existing methods compared to the proposed method for segmenting multi-panel images. The framework in [21] takes 20.7 seconds, and the method in [22] takes 1.1 seconds, while the approach in [23] does not report the processing time taken and is, therefore, indicated by “Nil”. ’ in the table. The proposed method takes 10.5 seconds. This indicates that our framework outperforms existing methods in segmentation of multi-panel images.

**Table 4:** Comparison based on sub-image separation in terms of average CPU time.

Methods	Sub-images segmentation(in seconds)
Framework [21]	20.7
Dynamic Programming [22]	1.1
Method [23]	Nil
Proposed method	**10.5**

### 3.4. Comparison with irregular image segmentation methods

This section presents a comparison of segmentation accuracy for irregular multi-panel images between our method and existing approaches [13, 19, 25] as follows. Table 5 reports that the multi-panel image segmentation methods [13, 19, 25] do not report the processing time taken by these methods to identify the type of input images and are, therefore, as indicated by “Nil.” in the table. In contrast, our approach has the potential to automatically classify input images into their respective types. It performs this task with remarkable efficiency, taking 8.10 seconds to identify a single-panel image, 16.75 seconds to identify regular multi-panel images, and 25.20 seconds to identify irregular multi-panel images. These results indicate that our framework outperforms existing methods in terms of CPU time in image type identification.

**Table 5:** Comparison based on average CPU time in image type identification.

Methods	Image type identification (in seconds)		
	Single—panel	Regular- compound	Irregular-compound
Framework [13]	Nil	Nil	Nil
Compound image segmentation [19]	Nil	Nil	Nil
Compound figure detection [25]	Nil	Nil	Nil
Proposed method	**8.10**	**16.75**	**25.20**

Table 6 highlights the accuracy comparison of our method against existing methods in image type identification. The methods [13, 19] do not consider the image type identification and are indicated by “Nil”. in the table. Similarly, the approach in [25] can dentify the type of input image as a multi-panel image with an average accuracy of 82.82%, but it does not report the accuracy of the identification of single panel images and, therefore, denoted by “Nil” in the table. Contrarily, our method performs the image type identification task with significant efficiency and accuracy of 96.10, 95.75, and 90.50 in the identification of single-panel images, regular compound, and irregular compound images, respectively. These results indicate that our method performs better compared to exiting methods in image type identification.

**Table 6:** Comparison based on accuracy in terms of percentage in image type identification.

Methods	Image type identification (in percentage)		
	Single—panel	Regular- compound	Irregular-compound
Framework [13]	Nil	Nil	Nil
Compound image segmentation [19]	Nil	Nil	Nil
Compound figure detection [25]	Nil	82.82	82.82
Proposed method	**96.10**	**95.75**	**90.50**

Table 7 represents the accuracy comparison between our method and existing methods during segmenting multi-panel images in sub-images. The method in [13, 19] achieves an accuracy with average precision of 96.64. The method in [13, 19] achieved segmentation accuracy with average precision of 79.37, average recall of 86.5, and average F1-measure of 86.51, respectively. However, the work in [25] was not aimed at image segmentation; it focused on identifying if an image is multi-panel. Contrarily, the proposed method accomplishes the task of multi-panel image segmentation with improved accuracy of 96.81 for average precision, 90.30 for average recall, and 93.10 for F1-measure. The findings indicate that our method is effective at segmenting sub-image panels from multi-panel images compared to other methods.

**Table 7:** Comparison of our method based on sub-image segmentation.

Methods	Sub-images segmentation(in percentage)		
	Average precision	Average recall	Average F1-measure
Framework [13]	96.64	Nil	Nil
Compound image segmentation [19]	79.37	86.51	86.51
Compound figure detection [25]	Nil	Nil	Nil
Proposed method	**96.81**	**90.30**	**93.10**

Table 8 presents a comparison of performance between our method and existing methods in terms of CPU time required in segmentation of multi-panel images into sub-images. The approach in [13, 19] takes an average of 0.74 seconds to segment each image in the dataset. Again, the work in [25] ‎did not undertake the task of image segmentation, thus its contribution is marked as Nil in the table to reflect its non-application to the segmentation tasks. In comparison, our proposed approach demonstrates outstanding efficiency, taking only 0.52 seconds on average to segment an image. The outcomes indicate that our framework outperforms exitings methods in CPU time efficiency for segmenting multi-panel images.

**Table 8:** Comparison on sub-image segmentation using average CPU time.

Methods	Sub-images segmentation
Framework [13]	0.74
Compound image segmentation [19]	0.74
Compound figure detection [25]	Nil
Proposed method	**0.52**

**Fig 10:**
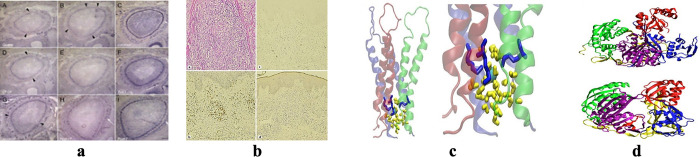
Examples of Successfully Segmented Images; (a, b) Regular Multi-panel Images, (c, d) Irregular Multi-panel Images. Reproduced with Permission from ImageCLEF 2016, and ImageCLEF 2014, ImageCLEF 2015.

Our multi-panel image segmentation approach achieves satisfactory results regarding both time efficiency and accuracy for multi-panel images that contain sub-image panels along horizontal and vertical directions, as well as those with visible space gaps between the sub-images. Fig 10 illustrates some successful results reported by the proposed method.

**Fig 11:**
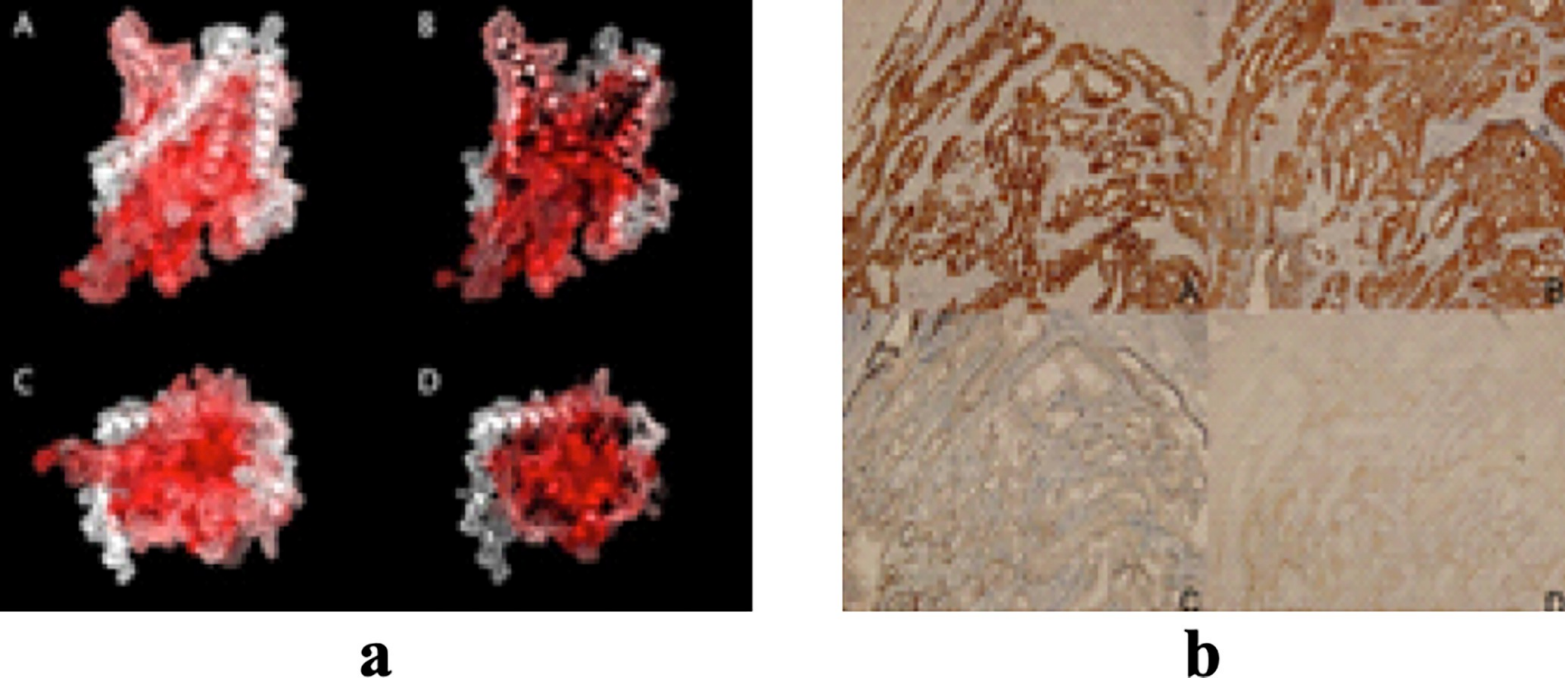
Examples of Multi-panel Images Not Effectively Handled by our Framework, (a) Multi-panel Image with Dark/Black Background, (b) Multi-panel Image With No Visible Background (images taken from [22]). Reproduced with Permission from ImageCLEF 2014, and ImageCLEF 2015.

However, the performance of our method in terms of accuracy for some specific multi-panel images is not satisfactory and highlights the need for further enhancements. This encounters challenges with multi-panel images that have dark (black) backgrounds or having no backgroud, as illustrasted in Fig 11. Our framework does not address these image types. In summary, our framework has effectively addressed the challenges presented by the previous approaches, achieving better accuracy in both image type identification and sub-image segmentation tasks.

## 4. Conclusion

This research introduces a hybrid multi-panel image segmentation method for medical images. It classifies images into single-panel, regular multi-panel, or irregular multi-panel categories, with further sub-image segmentations for multi-panel images in both regular and irregular categories. Tested on a dataset of 5750 medical images, our method shows improved efficiency and accuracy compared to existing approaches. As a future work, our goal is to develop more efficient approaches for segmenting multi-panel images and for the classification of the segmented sub-images according to their imaging modality, such as X-rays, MRIs, CT scans, and others.

By enhancing the segmentation accuracy and specifying imaging modality, we aim to significantly improve the performance of content-based medical image retrieval(CBMIR) systems. This will enable the CBMIR systems to deliever more precise results in response to specific image queries from doctors, thereby supporting the making of timely and accurate clinical decisions based on the medical information retrieved from medical image databases.
